# Survey on the indexes of health equity in the physical environment and infrastructures of Kermanshah province, Iran

**DOI:** 10.1186/s42506-021-00068-z

**Published:** 2021-04-01

**Authors:** Sohyla Reshadat, Shahram Saeidi, Alireza Zangeneh, Ali Almasi, Samira Rahimi Naderi, Ramin Teimouri, Raziyeh Teimouri, Kobra Gholami Kiaee, Mehdi Khezeli

**Affiliations:** 1 Social Development & Health Promotion Research Center, Health Institute, Kermanshah University of Medical Sciences, Kermanshah, Iran; 2 Department of Nursing, School of Nursing and Midwifery, Hamadan University of Medical Sciences, Hamadan, Iran; 3 Clinical Laboratory Science, Tabriz University, Tabriz, Iran; 4 Department of Art, Architecture and Design, University of South Australia, Adelaide, Australia; 5 Social Determinants in Health Promotion Research Center, Hormozgan Health Institute, Hormozgan University of Medical Sciences, Bandar Abbas, Iran

**Keywords:** Health equity, Environment, Index, Geographic Information Systems

## Abstract

**Background:**

Health equity is directly associated with the proper distribution of resources, the existence of infrastructures, and the balanced physical environment. The present study aimed to survey the indexes of health equity in the physical environment and infrastructures of Kermanshah province based on the national indexes.

**Results:**

The results revealed that access to transportation, health centers, solid waste management, and green and sports per capita had the least distance from the negative ideal whereas the noise pollution index had the greatest distance. However, house hygiene and air pollution indexes were within the negative and positive ideal ranges.

**Conclusions:**

The health equity indexes were not distributed equitably across counties and geographical regions of Kermanshah province.

##  Introduction

Nowadays, governments by providing health services and adequate and timely care as well as equitable distribution of services can affect the community’s well-being and health. It should be noted that providing the best healthcare alone is not sufficient [1]. Some experts define health equity as “lack of systematic differences in health means creating equal opportunities for health and reducing health differences to the lowest possible level” [2]. Health equity implies that all segments of the society should ideally have a fair chance of benefiting from services and financing to achieve their potential full health. Establishing health equity relates with health determinants and has special complexity due to numerous dimensions and effects, causing officials and managers in this regard to face the complex and major challenges. In response to these concerns, the Commission for Social Determinants of Health was established in the World Health Organization (WHO), through which some recommendations were presented to develop and monitor rights to good health at local, national, and international levels [3].

Recently, to assess gap in the health status of urban population and its determinants, a tool called “responsiveness and justice measurement in urban health” has been developed by the Health Development Center of the WHO which is used in Kobe, Japan [4, 5]. After designing this tool, the Tehran municipality announced to the WHO Regional Office for the Eastern Mediterranean (EMRO) its readness to investigate and measure health equity as a pilot study in Tehran in 2007. The outcome of the efforts of this working group incorporated the proposal of six areas instead of four areas within Iran, including infrastructure and physical environment, human and social development, economic development, governance, nutrition, and health [6].

One of the levels for national and regional planning is awareness of the capabilities of different provinces and their counties. Moreover, evidence has shown that Geographic Information System (GIS) has always been one of the most useful tools in this respect [7].

The destruction of the infrastructures of Kermanshah province because of the 8-year war between Iran and Iraq caused this province to fall behind development compared to other provinces. This issue led to the backwardness of this province and many other detrimental consequences including the settlement of a large part of war veterans of the province in the capital city of Kermanshah and its excessive population growth. The main impact of this trend was the interruption and failure in the system of service distribution, and subsequently led to the failure of citizens’ adequate access to such services [8]. On the other hand, Kermanshah is also faced with other problems such as poverty, high general fertility in some regions, AIDS, cancer [9], and economic loss in the areas of animal husbandry, agriculture, farming, and gardening due to air pollution (dust phenomenon).

Air pollution occurs in Kermanshah like other big cities, which ends to 500 as hazardous air quality that necessitates avoidance of outdoor activities. The Air Quality Index (AQI) is an important tool to assess air quality and evaluate its effects on health. AQI is determined by six major pollutants as important criteria: ozone (O_3_), sulfur dioxides (SO_2_), inhalable particles (PM_10_), fine particulate matter (PM_2.5_), nitrogen dioxides (NO_2_), and carbon monoxide (CO) [10]. The AQI scale is in the range of 0 to 500 to qualify the overall quality of the air into six levels in different colors (good in green, moderate in yellow, lightly polluted in orange, moderately polluted in red, heavily polluted in purple, and severely polluted in maroon). These levels indicate air pollution overall effects on human health and decent reference for people’s outdoor activities in a numerical pattern. A low number means good air quality, while an increasing number means worsening air quality, for example values over 300 represent hazardous air quality which is the health warning of emergency condition and everyone is more likely to be affected [11]. The present study aimed to survey the indexes of health equity in the physical environment and infrastructures of Kermanshah province based on the national indexes.

## Methods

### Design and setting

This cross-sectional study was performed using quantitative models, based on the national indexes codified by the Ministry of Health and Medical Education of Iran in 2016. The geographic area of this study was Kermanshah province, western Iran, and its statistical population was all of 14 counties including Kermanshah, Eslamabad-e Gharb, Paveh, Harsin, Kangavar, Sonqor, Javanrud, Ravansar, Gilan-e Gharb, Sahneh, Qasr-e Shirin, and Sarpol-e Zahab, Dalahu, and Salas-e Babajani.

Data was collected about eight indexes related to the assessment of equity in the area of physical environment and infrastructures, codified by the Ministry of Health and Medical Sciences [6] and based on the study conducted in Kobe, Japan [5]. These indexes were as follows:

1. The data on house hygiene (the percentage of households with waste disposal systems, sanitary toilets, and access to public drinking water network) were collected through the statistics provided by health center of Kermanshah.
2. The data on air pollution (dust) including the percentage of clean days in the year were collected from the meteorological organization of Kermanshah.
3. The data on noise pollution by industries were collected from the Industry, Mine and Trade Organization of Kermanshah province.
4. The data on access to public transportation was collected from the municipalities of counties.
5. The data on access to health centers (including primary health care).
6. The data on solid waste management (the status of waste management in urban areas, hospitals, and villages) was collected from the Health Network in each county.
7. The data on green space per capita in the province were collected from the municipalities of counties.
8. The data on sports per capita were collected from the Sport Department of Kermanshah province.

### Data collection

The required information was collected through documentary methods from the related departments in Kermanshah province. Additionally, the demographic data of each county were collected through the statistical blocks of the Official Statistics Center of Iran. Moreover, the latest population statistics (the demographic data of 2016) published by the Statistical Centre of Iran were used as the basis of the study.

### Data analysis

In the first step, data about the studied eight indexes were collected. Then, the Technique for Order of Preference by Similarity to Ideal Solution (TOPSIS) that is a multi-criteria decision analysis method was used. This technique, as one of the best multi-criteria decision-making models which are used extensively, was first proposed by Hawang and Yoon in 1981 [12]. In this method, m alternatives are evaluated by n criteria. This technique is based on the notion that the selected alternative should have the furthest distance from the negative ideal solution. It is assumed that the utility of each index is uniformly increasing or decreasing. Problem-solving using TOPSIS includes the following steps:

1. Formation of data matrixes based on an alternative (counties) and m indexes (the applied indexes in the research),
2. Creating the normalized decision matrix.
3. Establishing the weighted normalized matrix (V): the normalized matrix (N) is multiplied by the diagonal matrix of weights (Wn).
4. Determining the ideal positive solutions (determination of the maximum value for each of the weighted standardized indicators) and negative solutions (determination of the minimum value for each of the weighted standardized indicators).
5. Calculating the distance of each alternative from the positive ideals and negative ideals.
6. Determining the relative proximity of alternatives to the ideal solution.${\mathrm{Cl}}_{\mathrm{i}}=\frac{{\mathrm{d}}^{-}}{{\mathrm{d}}^{-}+{\mathrm{d}}^{+}}$
7. Ranking the alternatives in ascending order by the value of Consistency Index (CI): the alternatives selected must have the shortest distance from the positive ideal solution and the farthest from the negative ideal solution. If the CI value is closer to one, the situation is better.

Then, the priority map of counties was drawn in terms of the indexes of health equity using the Arc/GIS 10.6 Software.

It is worth noting that the Shannon’s Entropy Method was used to weighting in TOPSIS because knowing the relative weights of indexes is an effective step in the problem-solving process when it comes to multi-criteria decision making. Moreover, *K* is a positive constant, and the *P*-value (normalized matrix) was calculated for each i (alternative) and j (index). TOPSIS method was used in the TOPSIS-SOLVER Software. Finally, the results of eight indices were presented in proper maps to display the condition of counties using the Arc/GIS 10.6 Software.

## Results

**Fig. 1:**
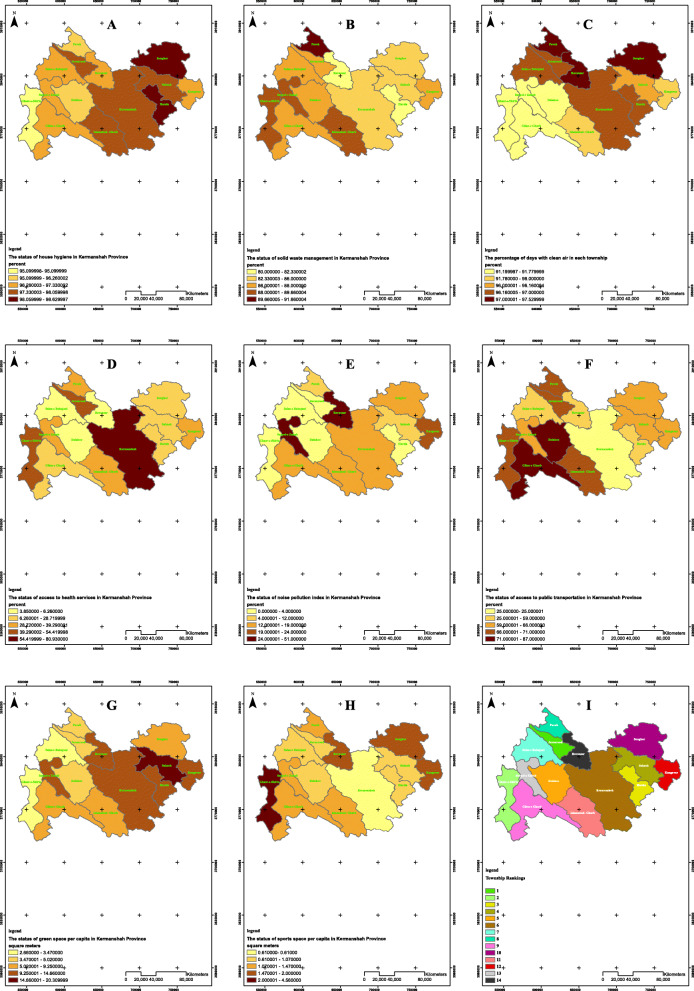
The map of the status of health equity in physical and infrastructure indexes and counties ranking in Kermanshah province in 2016 using GIS

As shown in Table 1 and Fig. 1a, all counties obtained further than 95% of scores in house hygiene index. All households residing in Kermanshah province had access to safe drinking water and standard sanitary toilets. Also, household sanitary sewage disposal was lower than 90% in only three cities, including Dalahu (88.8%), Paveh (87.5%), and Qasr-e Shirin (85.3%).

**Table 1:** The status of physical environment indexes, Kermanshah, Iran

	Number of households	The status of house hygiene (%)				The status of households’ solid waste management (%)				Percentage of clean days in the year in terms of dust phenomenon per household (%)	Status of access to health services per household in percentage (%)	Status of noise pollution per household in percentage (%)	Status of access to public transportation per household in percentage (%)	Green space per capita (in m^2^) per person	Sports per capita (in m^2^) per person
		Families with appropriate house hygiene (%)	Families with waste disposal systems (%)	Families with safe drinking water (%)	Families with sanitary toilets (%)	Urban areas (%)	Rural areas (%)	Hospitals (%)	Total (%)						
Islamabad-Gharb	40,052	100	100	93	97.66	90	78	100	89.33	96	33.6	19	69	7.51	1.23
Paveh	15,899	100	100	87.5	95.83	89	86	100	91.66	97.53	39.29	12	71	4.84	1.4
Salas-e Babajani	8811	100	100	91.9	97.33	85	77	100	87.33	97	3.85	0	50	3.47	1.07
Javanrood	17,830	100	100	94.2	98.06	88	73	100	87	96.71	54.42	0	69	4.08	0.9
Dalahoo	10,362	100	100	88.8	96.26	89	71	100	86.66	91.5	6.26	0	87	5.02	1.01
Ravansar	12,125	100	100	92	97.33	89	51	100	80	97.2	5.36	51	58	10.46	1.92
Sarpol-e Zahab	21,670	100	100	92	97.33	90	79	100	89.66	91.2	31	48	63	14.66	1.47
Songhor	26,189	100	100	95.1	98.36	91	67	100	86	97.5	28.72	17	62	7.54	1.74
Sahneh	22,046	100	100	93	97.66	92	66	100	86	96.16	22.67	8	65	20.31	0.91
Ghasr-e-Shirin	6050	100	100	85.3	95.1	87	80	100	89	91.78	46.2	11	70	2.66	4.56
Kermanshah	287,931	100	100	93.9	97.96	95	61	100	85.33	96.7	80.93	14	25	10.77	0.61
Kangavar	22,652	100	100	92	97.33	91	73	100	88	95.89	32.65	24	66	11.03	2
Gilan-e Gharb	15,617	100	100	93.2	97.33	86	77	100	87.66	91.78	27.6	14	86	9.25	1.24
Harsin	23,173	100	100	95.9	98.63	93	54	100	82.33	96.98	27.61	4	59	11.46	0.87
Whole province	530,407	100	100	92	97.32	89.64	70.92	100	86.85	95.28	31.44	15.85	64.28	8.79	1.49

Source: Water and Sewage Department of Kermanshah province; the Vice-chancellery for Health of Kermanshah province; Kermanshah Meteorological Department; Vice-chancellery for Health of Kermanshah province; Industry, Mine and Trade Organization of Kermanshah province; Statistics Center of Kermanshah City Hall; Municipality Organization and Kermanshah Sports and Youth Department, 2016

The results of solid waste management in urban areas showed that seven cities had provided more than 90% of the proper waste management. Table 1 and Fig. 1b showed that Salas-e Babajani and Kermanshah counties had the lowest and highest levels of urban proper waste management (85% and 95%, respectively). In the rural waste management index, only in Paveh County, more than 80% of proper waste management was provided, and in other cities, it was less than 70% (86% and 51% in Paveh and Ravansar, respectively). All counties had proper waste management in hospitals and inpatient treatment centers.

According to Table 2 and Fig. 1c, Paveh and Sarpol-e Zahab had the most and the fewest percentage of clean days, an indicator of lacking air pollution (97.53% vs. 91.2%, respectively).

**Table 2:** The indexes of equity in physical environment and infrastructures across Kermanshah province, Iran

Counties	House hygiene (%)	Air pollution (%)	Noise pollution from industries (%)	Access to public transportation (%)	Access to health centers (%)	Solid waste management (%)	Green space (m^2^)	Sports per capita (m^2^)
Kermanshah	97.96	96.7	14	25	80.93	85.33	10.77	0.61
Islamabad- Gharb	97.66	96	19	69	33.6	89.33	7.51	1.23
Harsin	98.63	96.98	4	59	27.61	82.33	11.46	0.87
Kangavar	97.33	95.89	24	66	32.65	88	11.03	2
Songhor	98.36	97.5	17	62	28.72	86	7.54	1.74
Sahneh	97.66	96.16	8	65	22.67	86	20.31	0.91
Javanrood	98.06	96.71	0	69	54.42	87	4.08	0.9
Sarpol-e Zahab	97.33	91.2	48	63	31	89.66	14.66	1.47
Paveh	95.83	97.53	12	71	39.29	91.66	4.84	1.4
Gilan-e Gharb	97.33	91.78	14	86	27.6	87.66	9.25	1.24
Ravansar	97.33	97.2	51	58	5.36	80	10.46	1.92
Dalahoo	96.26	91.5	0	87	6.26	86.66	5.02	1.01
Ghasr-e-Shirin	95.1	91.78	11	70	46.2	89	2.66	4.56
Salas-e Babajani	97.3	97	0	50	3.85	87.33	3.47	1.07

The results showed that access to health centers was more than 80% in only one county and less than 60% in other cities. Figure 1d shows that Kermanshah (80.93%) and Ravansar (5.36%) counties had the highest and least access to health centers, respectively. Moreover, Ravansar and Harsin according to Fig. 1e had the highest and lowest levels of noise pollution (51% and 0.4%, respectively). Furthermore, Dalahu (87%) and Kermanshah (25%) counties had the highest and least access to public transportation, respectively (Fig. 1f). According to Fig. 1g, Sarpol-e Zahab and Ghasr-e-Shirin had the highest and lowest green space per capita (14.66 and 2.66 m^2^, respectively), and Fig. 1h showed that Ghasr-e-Shirin (4.56 m^2^) and Kermanshah (0.61 m^2^) had the highest and lowest values of sports per capita.

The results demonstrated that Javanrood, Ghasr-e-shirin, Harsin, Sahneh, and Dalahoo were ranked one to five according to the TOPSIS. Regarding the counties of Kermanshah, Salas-e Babajani, Paveh, Gilan-e Gharb, and Songhor were ranked sixth to tenth, respectively. Moreover, the counties of Islamabad, Kangavar, Sarpol-e Zahab, and Ravansar held the 11th to 14th positions, respectively (Tables 2, 3, 4, 5, 6 and 7).

**Table 3:** Standardized matrix of indexes

Counties	House hygiene	Air pollution	Noise pollution from industries	Access to public transportation	Access to health centers	Solid waste management	Green space	Sports per capita
Kermanshah	0.2691	96.7	14	25	80.93	85.33	10.77	0.61
Islamabad-Gharb	0.2682	96	19	69	33.6	89.33	7.51	1.23
Harsin	0.2709	96.98	4	59	27.61	82.33	11.46	0.87
Kangavar	0.2673	95.89	24	66	32.65	88	11.03	2
Songhor	0.2702	97.5	17	62	28.72	86	7.54	1.74
Sahneh	0.2682	96.16	8	65	22.67	86	20.31	0.91
Javanrood	0.2693	96.71	0	69	54.42	87	4.08	0.9
Sarpol-e Zahab	0.2673	91.2	48	63	31	89.66	14.66	1.47
Paveh	0.2632	97.53	12	71	39.29	91.66	4.84	1.4
Gilan-e Gharb	0.2673	91.78	14	86	27.6	87.66	9.25	1.24
Ravansar	0.2673	97.2	51	58	5.36	80	10.46	1.92
Dalahoo	0.2644	91.5	0	87	6.26	86.66	5.02	1.01
Ghasr-e-Shirin	0.2612	91.78	11	70	46.2	89	2.66	4.56
Salas-e Babajani	0.2673	97	0	50	3.85	87.33	3.47	1.07
Shannon’s Entropy of weighting	0.0001	0.0003	0.4753	0.0287	0.207	0.0006	0.1378	0.1502

**Table 4:** Standardized weight matrix of indexes

Counties	House hygiene	Air pollution	Noise pollution from industries	Access to public transportation	Access to health centers	Solid waste management	Green space	Sports per capita
Kermanshah	0	0.0001	0.0803	0.0029	0.1206	0.0002	0.0398	0.0139
Islamabad-Gharb	0	0.0001	0.109	0.008	0.0501	0.0002	0.0278	0.028
Harsin	0	0.0001	0.0229	0.0069	0.0411	0.0002	0.0424	0.0198
Kangavar	0	0.0001	0.1376	0.0077	0.0487	0.0002	0.0408	0.0455
Songhor	0	0.0001	0.0975	0.0072	0.0428	0.0002	0.0279	0.0396
Sahneh	0	0.0001	0.0459	0.0076	0.0338	0.0002	0.0751	0.0207
Javanrood	0	0.0001	0	0.008	0.0811	0.0002	0.0151	0.0205
Sarpol-e Zahab	0	0.0001	0.2753	0.0073	0.0462	0.0002	0.0542	0.0334
Paveh	0	0.0001	0.0688	0.0083	0.0586	0.0002	0.0179	0.0318
Gilan-e Gharb	0	0.0001	0.0803	0.01	0.0411	0.0002	0.0342	0.0282
Ravansar	0	0.0001	0.2925	0.0068	0.008	0.0001	0.0387	0.0437
Dalahoo	0	0.0001	0	0.0101	0.0093	0.0002	0.0186	0.023
Ghasr-e-Shirin	0	0.0001	0.0631	0.0081	0.0689	0.0002	0.0098	0.1037
Salas-e Babajani	0	0.0001	0	0.0058	0.0057	0.0002	0.0128	0.0243

**Table 5:** Positive and negative ideals

Indexes of equity in the physical environment and infrastructures	Positive ideals (A+)	Negative ideals (A−)
House hygiene	0	0
Air pollution	0.0001	0.0001
Noise pollution from industries	0	0.2925
Access to public transportation	0.0101	0.0029
Access to health centers	0.1206	0.0057
Solid waste management	0.0002	0.0001
Green space	0.0751	0.0098
Sports per capita	0.1037	0.0139

**Table 6:** Distance from the positive and negative ideals

Indexes of equity in the physical environment and infrastructures	Positive ideals (A+)	Negative ideals (A−)
Kermanshah	0.1258	0.2432
Islamabad-Gharb	0.1576	0.1903
Harsin	0.1223	0.2739
Kangavar	0.1694	0.1667
Songhor	0.148	0.201
Sahneh	0.1286	0.2568
Javanrood	0.11	0.3022
Sarpol-e Zahab	0.2945	0.0656
Paveh	0.1305	0.2307
Gilan-e Gharb	0.1419	0.2171
Ravansar	0.3212	0.0417
Dalahoo	0.1487	0.2929
Ghasr-e-Shirin	0.1045	0.2544
Salas-e Babajani	0.1529	0.2927

**Table 7:** Township rankings using TOPSIS

Counties name	Relative proximity of alternatives (Cli)	Ranking
Javanrood	0.7332	1
Ghasr-e-Shirin	0.7088	2
Harsin	0.6913	3
Sahneh	0.6663	4
Dalahoo	0.6633	5
Kermanshah	0.6591	6
Salas-e Babajani	0.6568	7
Paveh	0.6387	8
Gilan-e Gharb	0.6047	9
Songhor	0.5759	10
Islamabad- Gharb	0.547	11
Kangavar	0.496	12
Sarpol-e Zahab	0.1822	13
Ravansar	0.1149	14

As shown in Table 5, comparison of 8 indicators showed that access to transportation (0.0029), access to health centers (0.0057), solid waste management (0.0001), green space per capita (0.0098), and sports per capita (0.0139) had the shortest distance with a negative ideal. On the other hand, the noise pollution index had the longest distance from the negative ideal (0.2925), and the house hygiene and air pollution indices were between the two negative and positive ideals.

## Discussion

This study aimed to survey the indexes of equity in the physical environment and infrastructures of Kermanshah province based on the national indexes.

The results showed that the indexes of access to transportation, access to health centers, solid waste management, and green and sports per capita had the least distance from the negative ideal whereas the noise pollution index had the greatest distance. However, house hygiene and air pollution indexes were within the negative and positive ideal ranges. Hence, it can be concluded that the status of equity in the physical environment and infrastructures in Kermanshah province was unbalanced.

Additionally, the findings of the present study on better conditions of eight indexes in some counties indicated the lack of sustainable development in Kermanshah province, even if the standard per capita was similar between counties. It is well known that any development that is not comprehensive and not following a systematic and integrated model cannot be sustainable [13]. The results of a study performed by Maleki et al. in Khuzestan province showed the lack of spatial equity in distribution of facilities and health services across its counties [13].

The results of the present study showed that the majority of households had proper house hygiene, the highest level was in Harsin and the lowest in Ghasr-e-Shirin, which was consistent with the results of a study conducted by Ghadermarzy et al. [14]. Given that Ghasr-e-Shirin is located in the border area of Iran with Iraq and was the first point attacked by Iraqi army in the 8-year Iran-Iraq war, it is likely that damages on its infrastructures, late reconstruction of destroyed houses, in addition the long distance from the province center have led to the lower level of house hygiene; however, this explanation requires further studies. House hygiene indicators such as toilets and sewage are important because they have a direct impact on the prevalence of infectious diseases and the physical health of family members [15, 16]. However, the general situation of the house hygiene index in the province was favorable.

The findings of the present study indicated that Kermanshah province had good category of Air Quality Index (AQI) around 95% of the days of the year. Kermanshah province is exposed to air pollution only 5% of the days of the year, showing a downward trend compared with the statistics of 2009–2010, similar to the results of a study performed by Shamshiri et al. [17]. In the last two decades, dust storms in the south and west of Iran have been a reemerging phenomenon whose number of days in previous years has been variable and affected by rainfall and air temperature [18, 19]. Due to the increase in rainfall over the past few years in Kermanshah province [20], this increase in rainfall has probably been effective in reducing dust storm and air pollution.

Our findings on solid waste management showed that all hospitals in the province had recycling and disposal waste facilities. Moreover, urban and rural areas had access to solid waste management. However, there is no integrated and codified program for waste management in urban and rural areas of Kermanshah province. Also rural areas of Ravansar and Harsin counties had the poorest solid waste management, requiring the attention and effort of health authorities of Kermanshah province. This finding was consistent with the results of a study conducted by Bakhtyari et al. [21]. Studies show that access to sound waste management services is still limited in developing countries, especially in rural areas [22]. Disposal of waste in the natural environment and the lack of dumpsites are the main reasons for the low quality of waste management in rural areas [23].

Other results showed that Kermanshah and Javanrood had the highest access to health centers while Salas-e Babajani, Ravansar, and Dalahu had the lowest access (less than 10%). This finding was consistent with the results of studies done by Sulaimany et al. [24] and Mousavi et al. [25]. This difference was very significant between the counties, which indicated a lack of geographical heterogeneity and inequality in access to health centers throughout Kermanshah province. This needs a rapid shifting in policies towards people’s access to health services. Similarly, health inequity between different regions of Iran [9] and within Kermanshah province in terms of access to health services have been shown [26]. Despite the primary health care system (PHC) at the level of health houses, rural and urban health centers, and hospitals in each county, it seems that the geographical condition, relative deprivation, and lack of road development in each county has affected the existence of health service centers and people’s access to the available health centers.

The results of our study showed the poor condition of sports per capita in Kermanshah province compared to the national [27] and the international standards [28]. This index was much lower in Kermanshah than provincial level. Similarly, the results of other studies have also pointed to the inappropriate distribution of sports spaces in other provinces of Iran [27, 29].

Another finding of present study indicated a significant shortage in the green space per capita in Kermanshah province compared to the global standards [30], and the value obtained is also lower than the national standards [31]. The lack of equal access to green space in the province was consistent with the results of a study conducted by Tajdar et al. [32]. The importance of access to green space has been emphasized in some other studies [6, 33].

Based on the results of the present study, more than 15% of the households in the province suffered from noise pollution. This statistic was calculated based on industrial-towns along with other structures [34]. The results of comparing counties demonstrated that Ravansar and Sarpol-e-Zahab had the highest and Sahneh and Harsin had the lowest percentage of noise pollution. This difference is probably due to the lack of suitability of land applications in Kermanshah province and other reasons such as the urban structure, as mentioned in other studies [34]. However, the results of this study showed that noise pollution is not limited to large and industrial cities and also exists in small cities of the province, which may be due to the activity of small technical business in the cites, development of urban housing complexes, and lack of proper land use. The problem of noise pollution is a public health challenge in all developed and developing countries, mainly due to roads, airports, industrial towns, and technical occupations [35, 36]. The negative effects of noise pollution in the counties can be mitigated through applying land use plans and creating audio maps and comprehensive analysis of various policies at the county level.

Our findings showed that more than half of the province’s population had appropriate access to public transportation. The results also indicated that Dalahu and Kermanshah counties had the highest and lowest percentages of access to public transportation, respectively, consistent with the results of study by Tajdar et al. which confirmed the differences in various regions of the province [32]. It is likely that the sudden increase in the population of Kermanshah city during the last two decades and the incompatibility of public transportation with the population is the main reason for this difference. That is why the urban Kermanshah monorail project was put on the agenda, but its slow progress contributed to the continued problem of public transportation in Kermanshah.

In the present study, consistent with the similar studies [32], we used the GIS to assess the environmental and physical infrastructural indexes. This indicates that geography and GIS can be applicable scientific tools in health and inequalities assessments. We also used Shannon’s Entropy Method and TOPSIS for weighting and ranking. Moreover, the Arc/GIS software was used for displaying the conditions of counties similar to some other studies [37].

The results of our study demonstrated that the counties of Islamabad, Kangavar, Sarpol-e-Zahab, and Ravansar were far from the positive ideal and had the lowest ranks in terms of health equity in physical environment and infrastructure. In addition to the necessity to investigate the causes of this inequity, there is a need for scientific planning and practical measures to reduce the inequalities. This issue has become more important due to the 7.3 magnitude earthquake in 2017 in the west of Kermanshah province, especially Sarpolzahab, Islamabad, and Ravansar, which had destructive effects on physical infrastructure and health.

The imbalance in the physical environment and infrastructures in Kermanshah province means that the implemented policies have not led to health equity in the province. In studies conducted outside Iran, policy-making and planning have also been mentioned as important issues on health equity [38, 39]. Concentration of services in urban places created bipolar areas and also led to migration from the rural to urban areas, and subsequently creating the inequity and misdistribution of services and facilities in the province. Imbalance and inequality in infrastructure is evident when we compare the counties. Javanrood and Ravansar, as two neighboring counties, have the highest and lowest rank in terms of equity in the physical environment and infrastructures, respectively. Similarly, Rezaei et al. concluded that Javanrood and Ravansar were developed and underdeveloped in terms of access to health services, respectively [40]. This finding indicates the need to precise regional planning in Kermanshah province towards balanced development in all areas.

### Study limitations

The present study only examined the indices of physical environment and infrastructures, and the reasons for this inequity were not assessed. Hence, it is recommended that the causes of this inequity be investigated for each of the eight indexes in future studies.

## Conclusion

The results of the present study revealed that the indices of health equity in physical environment and infrastructures were not distributed equitably between the counties of Kermanshah province. Accordingly, the asymmetric distribution of indexes in counties was the key factor in creating health inequity and should be considered as a challenge by planners, health managers, and policymakers. The model proposed in the present study can be used in all provinces of Iran and other developing countries.

## Data Availability

Data will be available upon request.
